# Practice and Barriers toward Breast Self-Examination among Palestinian Women in Gaza City, Palestine

**DOI:** 10.1155/2020/7484631

**Published:** 2020-03-30

**Authors:** Suha Baloushah, Waliu Jawula Salisu, Aymen Elsous, Maryam Muhammad Ibrahim, Fadia Jouda, Hanan Elmodallal, Zahra Behboodi Moghadam

**Affiliations:** ^1^Department of Reproductive Health, School of Nursing and Midwifery, Tehran University of Medical Science, Tehran 1419733171, Iran; ^2^Department of Medical-Surgical Nursing, School of Nursing and Midwifery, Tehran University of Medical Sciences, Tehran 1419733171, Iran; ^3^Faculty of Medical Sciences, Israa University-Gaza, Gaza Strip, State of Palestine; ^4^Unit of Planning and Policy Formulation, Ministry of Health, Gaza Strip 79702, State of Palestine; ^5^Palestinian Ministry of Health, Gaza Strip 79702, State of Palestine

## Abstract

Breast self-examination (BSE) is one of many first-line screening practices aimed at early detection and prevention of fatal outcomes of breast cancer. The present study aimed to identify Palestinian women's practices, awareness, and barriers to BSE. Using descriptive-analytical methods, a previously validated questionnaire was administered to a conveniently selected sample of women. The study was conducted among women who visited primary health clinics (PHCs) in Gaza City. A total of 390 participants who met the selection criteria answered the self-administered questionnaires. We used SPSS version 24.0 to analyze the data. The findings suggest that the practice of BSE among women in Gaza is low, with only 40% of the study participants reporting that they never practiced BSE before, even though 76.7% reported that they were aware of BSE. In general, the main barriers to BSE practices were that participants had wrong perceptions and lacked knowledge about BSE. Others also reported fear of detecting breast cancer as a barrier. The practice of BSE among Palestinian women in the Gaza Strip is low and marred with trivial issues as barriers that could be eliminated with dedicated and extensive educational campaigns in the area.

## 1. Introduction

Breast cancer (BC) is the leading cause of cancer mortality among women in the world [1, 2], accounting for 25.1% of all cancers [3]. However, its incidence in developed countries is high, while relative mortality is most considerable in less developed countries [3]. In Palestine, BC is the leading cause of cancer-related deaths among women: its mortality rate is 9.8% [4]. In the Gaza Strip alone, it accounts for 31.3% of all reported cancers occurring among Palestinian women with a prevalence of 149.1 per 100.000. BSE is considered one of the screening methods in addition to clinical breast examination and X-ray mammography that are aimed at early detection of BC [5]. The American Cancer Society recommended education and early commencement of BSE by the age of 20 years [6]. BSE is a patient-centered, inexpensive, and noninvasive method of screening for BC, which improves the chances of early BC detection [7, 8]. Statistics on the practice of BSE remains low in many countries. For example, it was reported in a study conducted in Kuwait that 12% practiced BSE [9].

Similarly, in Nigeria, 18.1% was reported [10]. Previous studies revealed several barriers to the practice of BSE, such as a previous family history of BC and the lack of knowledge about the practice of BSE [11–13]. There exists a dearth of literature on the practices and barriers to BSE among women of reproductive age in Palestine.

The present study aimed to identify the practice attitudes, awareness, and barriers of BSE among Palestinian women of reproductive age in Gaza City.

## 2. Materials and Methods

### 2.1. Study Design and Setting

This descriptive-analytical study was conducted at three selected PHCs out of six centers that provide a variety of primary health care services for women of all age groups in Gaza City of Palestine. The centers were selected purposively to include the ones with the highest attendance to ensure unrestricted participation and to capture a wide variety of the populace. The selected centers were Alzaytoun Center, Remal Martyr's Center, and Alfalah Center.

### 2.2. Study Sample and Sampling

A convenience sampling method was used from March 2017 to Jun 2017 to recruit 390 women who attended a maternal and child health clinic at any of the selected PHCs. The inclusion criterion was women aged 18 years and above, irrespective of their marital status. Three of the authors administered the questionnaires to women who reported to any of the three centers and willing to participate in the study. The women were approached to be included in the study as and when they arrived at the center for maternal and child health care services. The answered questionnaires were entered into SPSS immediately to avoid errors and for future analysis.

### 2.3. Study Instrument

In this present study, we used a self-administered questionnaire, which was developed based on extensive literature review and previous literature [14–16]. The validity and reliability were confirmed. The internal consistency was measured with Cronbach coefficient alpha and was <0.7. The content validity was measured using the conventional approaches of assessing both the item content validity index (I-CVI) and the scale content validity index (S-CVI). The I-CVI involves the measurement of the content validity of individual items while the S-CVI involves measuring the content validity of the overall scale. After the assessment, the results of the I-CVI and S-CVI showed to be above the recommended cut-offs. The questionnaire consisted of four parts: the first part sought participant's sociodemographic characteristics; the second part aimed to understand the participant's awareness and knowledge of breast cancer; BSE practices and reasons for performing BSE was the third part; and barriers toward performing BSE constituted the final part.

### 2.4. Sample Size

We used the traditional formula for calculating the sample size of cross-sectional studies [17] and an estimated sample size of 385 with a margin of error of 5% and confidence level of 95%. The sample size was increased by five, to 390 to overcome issues of missing data and nonresponse. In the end, all the participants that we invited to participate in the study responded; however, some parts of the questionnaires were left unanswered.

### 2.5. Data Analysis

The analysis was performed using SPSS version 24.0. Descriptive and analytical statistics were applied to all the variables in the study. Chi-Square test and simple logistic regression were used to test for the association between BSE practice and other categorical variables in the study. Bivariate binary logistic regression analysis was conducted to identify potential barriers to the practice of BSE. Findings were presented as crude odds ratios and 95% CI. All independent variables with *P* value <0.05 were considered statistically significant and chosen for logistic regression analysis. In the logistic regression, independent variables with *P* < 0.05 were noted as the main barriers to BSE.

### 2.6. Ethical Approval

We obtained ethical approval for this study from the Health Research Department of the Palestinian Ministry of Health.

## 3. Results

### 3.1. Characteristics of Respondents

A significant number of study participants, 340 (87.7%), were married, and most, 221 (56.7%), were below 30 years old. The majority, 369 (94.6%), reported that they have never suffered any breast disease in the past; 345 (88.5%) did not have a family history of BC as presented in Table 1.

### 3.2. Awareness of BC and BSE

There was a high level of awareness of BC and BSE among a good number of the participants, accounting for 352 (90.3%) and 299 (76.7%), respectively. The majority of the participants, 359 (92.1%), agreed that BSE was essential to them, yet, only 179 (45.9%) could perform BSE appropriately. Health care providers were the source of information regarding BSE to 138 (46.2%) of the participants; see Table 2.

### 3.3. Practice of BSE

Most of the study participants, 235(60.0%), had never practiced BSE Table 3. In total, 155 (40%) confirmed they had never performed BSE before; 115 (74.2%) of them indicated they first performed BSE after age 20. Only 27 (17.4%) reported they practiced BSE monthly. Of this, 94 (60.6%) performed it using three fingers, while 54 (34.8%) spend within 3–5 minutes performing BSE. Out of the 155 participants who confirmed they had never performed BSE before, 130 (83.9%) reported that they perform BSE for purposes of early detection. In total, 235 (60%) of the study participants do not perform the BSE.

### 3.4. Barriers to BSE

Regarding the barriers to the practice of BSE, 94 (40%) had the perception that they had no disease and therefore did not need a BSE. Other barriers included the lack of knowledge, 89 (37.9%); the fear of detecting cancer, 23 (9.8%); not thinking it is necessary, 15 (6.4%); the perception that it is time consuming, 6 (2.5%); the feeling that it deviates privacy, 6 (2.5%); and the feeling of embarrassment, 2 (0.9%); see Table 4.

### 3.5. Multivariate Analysis to Predict Barriers to BSE

The study findings showed that the respondents with a family history of BC (60%) practiced BSE more frequently than their counterparts who do not have any family history of BC (37.2%) (OR: 2.543, CI = 1.347–4.799, *P*=0.003), see Table 5. Also, those respondents with the previous history of exposure to breast disease (5.4%) practiced BSE more than those who did not have any previous history of exposure (94.6%) (OR: 2.59, CI: 1.05–6.42, *P*=0.033). Participants aged over 35 years (59.7%) practiced BSE more than those who were 35 years or less (33.1%) (OR: 0.33, CI: 0.20–0.53, *P*=0.000). The income status of participants directly affected their practice of BSE, with those earning a monthly income of 1500NIS or more practicing BSE more frequently (51.9%) than earners of 1500NIS or less (33.5%) (OR: 0.46, CI: 0.30–0.72, *P*=0.000). A logistic regression analysis was employed to identify the determining factors associated with BSE. After controlling other factors, only three factors were statistically significant with BSE; see Table 6.

Respondent's age, income, and previous family history of BC were the variables entered into multiple logistic regressions. BSE is more likely to be performed among respondents who are more than 35 years old (OR: 0.36, CI: 0.22–0.59, *P*=0.000) or have a family history of BC (OR: 2.36, CI: 1.22–4.85, *P*=0.01) and income more than 1500NIS per month (OR: 53, CI: 0.43–0.84, *P*=0.007).

## 4. Discussion

The BSE is an effective and cost-free technique to detect breast anomalies, which occasionally result in BC [18]. In low resourced areas such as the Gaza Strip in Palestine, access to advanced imaging technologies is a challenge. The BSE serves as an essential alternative and is critical in the early detection of breast tumors. The current study explored the awareness, practice, and barriers toward BSE among Palestinian Women in the Gaza Strip, Palestine. Perhaps, due to the destructive nature of breast cancer, most of the participants were much aware of the disease and the practice of self-breast examination. This is similar to findings reported by previous studies [19–21]. This finding may be inferred to the characteristic of our study participants which is young and educated.

Around 40% of respondents in this study practiced BSE; however, only 17.4% of them do so monthly. Even though the majority of the participants were aware of the BSE, only a few of them knew the techniques of BSE. Most women, as reported in earlier studies [16, 22], are often aware of cancer screening methods yet do not practice [23]. The possible explanation of the minimal rate of the regular monthly practice of BSE is the fewer rate of the previous history of exposure to breast disease among our study participants which is 5.4%. As is proved in the literature, women with a history of breast disease practice BSE more than their counterparts with no history of the disease [24].

As per the findings of this current study, the primary source of information about BSE to most of the participants is care providers (46.2%), followed by the Internet (22.5%), friends (21.5%), TV (19.4%), and radio (9.7%). In a similar study, TV and radio were the primary sources of information to 95.5% of the study participants [25], contrary to health care providers which we found in this study.

In this study, we identified many barriers for practicing BSE such as the perception of no disease threat, the lack of knowledge, the fear of detecting cancer, among others, that prevented participants from performing the BSE. These could have led to the low practice attitude among most of the participants who reported they had never practiced the BSE. However, it is interesting to note that a more significant proportion (83.9%) of the participants who practiced BSE did so with the intention of early detection, while only a few (17.4%) practiced monthly. These findings are congruent with that of previous studies which identified similar barriers such as lack of knowledge, lack of symptoms, fear of detecting cancer, lack of time, and lack of privacy among Malaysian women [7, 16], Emirati university students [26], and Jordanian female university students [27]. In another study conducted in Iran [28], Naghibi and colleagues reported that negative sociocultural behaviors contributed to the nonperformance of BSE. Society and cultural norms have often influenced women's health-seeking behaviors negatively. For example, Elobaid and colleagues, in their study, which was conducted in the United Arab Emirates, explored the factors influencing delays in presentation and health-seeking behaviors of BC patients. They reported that culture is keen on the decision-making process among BC patients [29]. The identified barriers in this current study are widespread and not specific to the current study setting.

Similar to previous studies [9, 30], we found that most of the participants had no family history of BC or previous exposure to breast disease. Although a first-line family history of BC is widely known as a strong predisposing factor [31], a growing body of evidence suggests that environmental factors [32] and lifestyle [33] are equally critical. Concerning this, it is difficult to exclude a population based on a single predisposing factor such as family history. In line with this, we believe that although most of the participants in this study had no family history, it does not exclude them from the risk zone of developing BC later in the future. Further analysis of our results, however, shows that the majority of the participants with a family history of BC practiced BSE more frequently than those without any family history. This is consistent with results of previous studies [7, 34]. Such occurrence is not surprising because once a family member is affected by a disease such as breast cancer, it is only reasonable that others take caution. In our investigation, age is one predictor of BSE. Women with the age of 40 years and above practiced BSE more frequently than those with the age lower than 40, which is in line with a previous study [35]. Working status and income limits play an essential role in access to the various resources of information regarding BC screening practice. In this study, family income is the third predictor. The study suggests that women with higher incomes were more likely to perform BSE than other women, which is consistent with the results of similar studies [36, 37].

The BSE as a primary preventive method is widely recommended as a way of early detection and, subsequently, early treatment [38]. It is therefore vital that efforts be put in place to ensure that women are encouraged, supported, and taught how to properly examine themselves in a quest for early detection of tumors. As well, policymakers must work tirelessly to put stringent measures in place to overcome the barriers outlined in this study. The early detection of BC followed by prompt treatment significantly reduces morbidity [7, 21]; hence, any obstacle to early detection must be encountered with urgency.

## 5. Conclusions

Although health care providers are the primary source of information about BSE, the practice among Palestinian women in the Gaza Strip is low and marred with trivial issues as barriers. These could be eliminated with dedicated and extensive educational campaigns in the area. The Palestinian Ministry of Health should lead these campaigns in teaching women how to effectively perform BSE and related benefits through such sources of information as TV, Radio, and the Internet due to the widespread access of these media outlets by a significant number of the target population.

## Figures and Tables

**Table 1:** Biographical data and cancer history.

Items	Categories	Frequency (%)
Participant's age	18–30	221 (56.7%)
	31–40	119 (30.5%)
	41–65	50 (12.8%)

Occupation	Have a job	170 (43.6%)
	Jobless	220 (56.4%)

Education	Primary	48 (12.3%)
	Secondary	193 (49.5%)
	University	138 (35.4%)
	Postgraduate	11 (2.8%)

Marital status	Single	40 (10.3%)
	Married	340 (87.2%)
	Others	10 (2.5%)

Income	Financial aid	162 (41.5%)
	1000–2000	160 (41.0%)
	2000–3000	42 (10.8%)
	More than 3000	26 (6.7%)

Family history of BC	Yes	45 (11.5%)
	No	345 (88.5%)

Previous exposure to breast disease	Yes	21 (5.4%)
	No	369 (94.6%)

**Table 2:** Awareness of breast cancer and knowledge of BSE.

Variables	Categories	Frequency (%)
Have ever heard about breast cancer	Yes	352 (90.3%)
	No	38 (9.7%)
Have ever heard about breast self-examination	Yes	299 (76.7%)
	No	91 (23.3%)
Knows how to do breast self-examination	Yes	179 (45.9%)
	No	211 (54.1%)
Breast self-examination is important	Yes	359 (92.1%)
	No	31 (7.9%)

Source of information (*n* = 299)		
TV	Yes	58 (19.4)
	No	241 (80.6%)
Radio	Yes	30 (9.7%)
	No	269 (90.3%)
Internet	Yes	67 (22.5%)
	No	232 (77.5%)
Friend	Yes	64 (21.5%)
	No	235 (78.5%)
Health care provider	Yes	138 (46.2%)
	No	161 (53.8%)

**Table 3:** Respondents practice and reasons for doing BSE.

Serial	Items	Categories	Frequency (%)
1	Have you ever performed BSE?	Yes	155 (40.0%)
		No	235 (60.0%)
	If yes (*n* = 155)		

2	Age of initial BSE	20 or less	40 (25.8%)
		More than 20	115 (74.2%)

3	Pattern of BSE	Monthly	27 (17.4%)
		Once in two months	15 (9.7%)
		Once in 3–5 months	16 (10.3%)
		Once a year	11 (7.1%)
		Irregularly	86 (55.5%)

4	How is BSE performed?	By using one finger	17 (11%)
		By using three fingers and palms of the hand	94 (60.6%)
		No specific methods	44 (28.4%)

5	How much time do you spend during the BSE?	Equal to or less than 3 minutes	85 (54.84%)
		3–5 minutes	54 (34.84%)
		More than five minutes	16 (10.32%)

6	Reasons for regular BSE	Early detection	130 (83.9%)
		Because there is a previous history of breast cancer	22 (14.2%)
		Other reasons	3 (1.9%)

**Table 4:** Barriers toward performing BSE.

Respondent's practice and reasons for performing BSE	Frequency (%)
Lack of knowledge	89 (37.9%)
It needs time	6 (2.5%)
I do not have a disease that requires breast self-examination	94 (40%)
Fear of detecting cancer	23 (9.8%)
I do not think it is necessary	15 (6.4%)
It deviates my privacy	6 (2.5%)
It is embarrassing	2 (0.9%)

**Table 5:** Bivariate analysis: association between breast self-examination (BSE) and sociodemographic variables.

	Variable		Practicing BSE	Not practicing BSE	OR	CI	*P* value
1	Age	40 and less	122	218	0.288	0.154–0.539	0.000^*∗*^^†‡§^
		More than 40	33	17			
2	Employment	Have a job	52 (43%)	69 (57%)	1.251	0.785–1.878	0.382^‡§^
		Jobless	103 (38.3%)	166 (61.7%)			
3	Income	1500 or less	87 (33.6%)	172 (66.4%)	0.469	0.305–0.720	0.000^*∗*^^†‡§^
		More than 1500	68 (51.9%)	63 (48.1%)			
4	Marital status	Married	140 (41.2%)	200 (58.8%)	0.612	0.322–1.164	0.132^‡§^
		Other	15 (30%)	35 (70%)			
5	Education	Primary and secondary	29	41	1.089	0.644–1.842	0.75^‡§^
		University and higher education	126	194			
6	Family history of BC	Yes	27 (60%)	18 (40%)	2.543	1.347–4.799	0.003^*∗*^^‡§^
		No	128 (37.1%)	217 (62.9)			
7	Previous exposure to breast disease	Yes	13 (61.9%)	8 (38.1%)		1.051–6.424	0.033^*∗*^^‡§^
		No	142 (38.5%)	227 (61.5%)	2.598		
8	Family history of cancer	Yes	40 (46%)	47 (54%)	1.391	0.860–2.251	0.178^§‡^
		No	115 (38%)	188 (62%)			

*P* value <0.05^*∗*^, *P* value <0.001^†^, Pearson Chi-Square^‡^, and Fisher's Exact Test^§^.

**Table 6:** Binary logistic regression of intimate partner violence with associated risk factors.

	Variable		*P* value	AOR	CI	
					Lower	Upper
1	Family history of BC	Yes	0.019^*∗*^	0.45	0.34	0.83
		No^†^	—	Ref	—	—
2	Age	More than 40	0.001^*∗*^	0.32	0.16	1.18
		40 and less^†^	—	Ref	—	—
3	Income	More than 1500 NIS	0.006^*∗*^	0.53	0.34	0.83
		1500 or less^†^ NIS	—	Ref	—	—

*P* < 0.05^*∗*^, reference category^†^, AOR: adjusted odds ratio, CI: confidence interval.

## Data Availability

The data used to support the findings of this study are available from the corresponding author upon request.
